# Breast Imaging Reporting and Data System (BI-RADS®): a success history and particularities of its use in Brazil

**DOI:** 10.61622/rbgo/2024AR06

**Published:** 2024-03-15

**Authors:** Vanessa Merjane, Douglas Marcel Puricelli Perin, Patrícia Martins Gomes El Bacha, Beatriz Medicis Maranhão Miranda, Almir Galvão Vieira Bitencourt, Wagner Iared

**Affiliations:** 1 Universidade Federal de São Paul São Paulo SP Brazil Universidade Federal de São Paulo, São Paulo, SP, Brazil.; 2 Hospital BP - A Beneficência Portuguesa de São Paulo São Paulo SP Brazil Hospital BP - A Beneficência Portuguesa de São Paulo, São Paulo, SP, Brazil.; 3 Grupo Dasa São Paulo SP Brazil Grupo Dasa, São Paulo, SP, Brazil.; 4 Radboud University Medical Center Nimegue Netherlands Radboud University Medical Center, Nimegue, Netherlands.; 5 Oncoclínicas Belo Horizonte MG Brazil Oncoclínicas, Belo Horizonte, MG, Brazil.; 6 Lucilo Maranhão Diagnósticos Recife PE Brazil Lucilo Maranhão Diagnósticos, Recife, PE, Brazil.; 7 Instituto de Medicina Integral Prof. Fernando Figueira Recife PE Brazil Instituto de Medicina Integral Prof. Fernando Figueira, Recife, PE, Brazil.; 8 Hospital A.C. Camargo Cancer Center São Paulo SP Brazil Hospital A.C. Camargo Cancer Center, São Paulo, SP, Brazil.

**Keywords:** BI-RADS®, History, Breast neoplasms, Screening, Brazil

## Abstract

BI-RADS® is a standardization system for breast imaging reports and results created by the American College of Radiology to initially address the lack of uniformity in mammography reporting. The system consists of a lexicon of descriptors, a reporting structure with final categories and recommended management, and a structure for data collection and auditing. It is accepted worldwide by all specialties involved in the care of breast diseases. Its implementation is related to the Mammography Quality Standards Act initiative in the United States (1992) and breast cancer screening. After its initial creation in 1993, four additional editions were published in 1995, 1998, 2003 and 2013. It is adopted in several countries around the world and has been translated into 6 languages. Successful breast cancer screening programs in high-income countries can be attributed in part to the widespread use of BI-RADS®. This success led to the development of similar classification systems for other organs (e.g., lung, liver, thyroid, ovaries, colon). In 1998, the structured report model was adopted in Brazil. This article highlights the pioneering and successful role of BI-RADS®, created by ACR 30 years ago, on the eve of publishing its sixth edition, which has evolved into a comprehensive quality assurance tool for multiple imaging modalities. And, especially, it contextualizes the importance of recognizing how we are using BI-RADS® in Brazil, from its implementation to the present day, with a focus on breast cancer screening.

## Introduction

BI-RADS® is an acronym for Breast Imaging Reporting and Data System. It is a worldwide example of standardization of imaging reports and results, ensuring organization, clarity, and efficiency. It allows for better communication, reduces confusion in the description and interpretation of images, facilitates result monitoring, provides agility in statistical studies, and is useful for conducting audits.^(1,2)^

BI-RADS® initiative arose to solve the lack of standardization and uniformity in mammography reports. The system is named as such because it contains three main components, including: 1) a lexicon of descriptors, 2) a reporting structure with final categories and recommended management, and 3) a structure for data collection and audits. An important component of this system is the lexicon, a dictionary of specific descriptors that were initially identified as predictive of benign and malignant disease, based on evidence from the literature. Once established, the lexicon provided new opportunities for quality assurance, more effective communication, research development, and better patient care. It is worth noting that the final evaluation categories of the lexicon are useful predictors of malignancy.^(1–4)^

It was developed by the American College of Radiology (ACR) and globally accepted by all specialties involved in breast pathology care. The system was developed by consensus among a group of experts in each section (subcommittees) and can be updated periodically to improve diagnostic parameters.

BI-RADS® was initially structured to be used in mammography reports, and is now also used in breast ultrasound, magnetic resonance imaging, tomosynthesis, and contrast mammography, demonstrating that the process of adapting to new technologies is always ongoing. There is a chapter dedicated to follow-up and result monitoring, including clinically relevant basic audits. Since its creation, five versions have been published, and the sixth edition is currently under development, validating 30 years of a history of pioneering and success.

The success of BI-RADS® guided the ACR to use the Reporting and Data System (RADS) model for other organs, including prostate (PI-RADS®), liver (LI-RADS®), thyroid (TI-RADS®), lung (LUNG-RADS®), head and neck (NI-RADS®), ovaries (O-RADS®), colon (C-RADS®), and bone (Bone-RADS®).^(5,6)^

**Figure 1:**
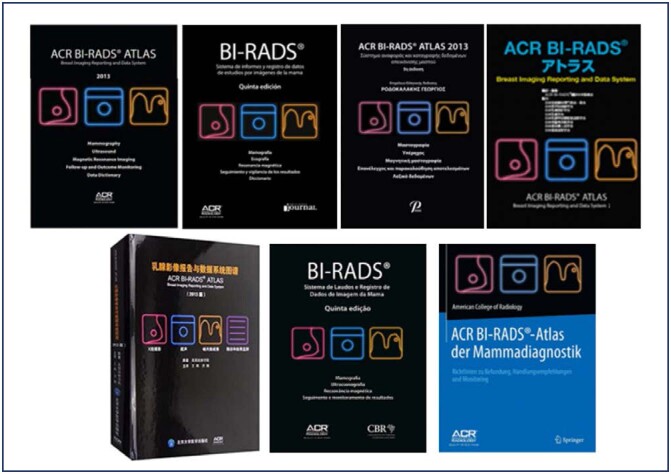
Original BI-RADS® Atlas in English and translations

There is no official list of countries that use the BI-RADS® classification system. However, after its creation in the United States, the system has already been adopted by countries on five continents, such as France, Germany, Italy, Spain, Netherlands, Greece, Canada, Mexico, Australia, New Zealand, Japan, China, among others. Some Latin American countries, in addition to Brazil, also use the system, such as Chile, Uruguay, Argentina, Peru. As seen in the figure 1, the system has been translated into 6 languages: Spanish, German, Portuguese, Mandarin, Japanese, and Greek.^(7)^

The purpose of this article is to provide a historical analysis of BI-RADS® from its early implementation to the latest status and reports on its introduction in Brazil while contextualizing its application in the country’s reality.

### Historical analysis of BI-RADS®

The American Cancer Society began recommending annual screening mammography in 1985. With the increased use of mammography, it became evident that there was variability in practices (radiation dose, image quality) and a lack of uniformity in mammography reports, making it difficult to guide patient management and publish results.^(2)^

In 1986, the American College of Radiology (ACR) convened a committee of radiologists, medical physicists, and a representative from the Food and Drug Administration (FDA) to develop a voluntary accreditation program for mammography and quality programs. The four goals of accreditation were: establishing quality standards, providing a mechanism for comparing performance with benchmarks, encouraging quality assurance practices and reproducibility, and low-dose screening mammography.^(2,8)^

The ACR recognized that clear descriptor terms and accurate communication of recommendations in mammography reports were important parts of a quality assurance program. Thus, in 1992, members of various ACR committees in a joint effort with other North American entities such as the American Medical Association (AMA), National Cancer Institute (NCI), Centers for Disease Control and Prevention (CDC), Food and Drug Administration (FDA), American College of Surgeons (ACS), and College of American Pathologists (CAP) developed the Breast Imaging Reporting and Data System (BI-RADS®) classification, aiming to standardize mammography reports.^(9)^ The involvement of different stakeholders in the development process helped promote consensus and facilitated acceptance. The BI-RADS® Committee went beyond advocating for the use of clear and standardized terms and recommended that mammography be "decision-oriented".^(2)^

Due in large part to concerns about patient safety and the quality of mammography screening, the ACR and the United States Congress sought to implement legislation to regulate mammography screening at the federal level. The intent of this legislation was to establish minimum standards that ensured all women had access to quality mammography services. The Mammography Quality Standards Act (MQSA) became law on October 27, 1992 (PL 102-539).^(10)^ The MQSA required the Department of Health and Human Services (HHS) to develop standards that would be applied through rigorous accreditation, certification, and inspection of equipment and personnel in mammography facilities. The Food and Drug Administration (FDA) was charged with implementing federal regulations that were published in October 1997 and are used to establish and enforce such procedures. The ACR is one of the four accreditation bodies approved by the FDA. The MQSA also encouraged all interpreting physicians to review their performance compared to benchmarks established by the Agency for Health Policy and Research. The BI-RADS® assessment categories proved to be a unique resource for measuring and improving the quality of mammographic interpretation. In the 2005 report "Improving Breast Imaging Quality Standards," the Institute of Medicine^(11)^ recognized that the BI-RADS® assessment provides an important tool for defining mammography positivity and negativity for interpretive performance auditing. Additionally, the report stated that the auditing requirements under MQSA are inadequate for measuring or improving interpretive quality and recommended that, to achieve this improvement, an expanded audit compatible with BI-RADS® should be mandatory.^(8)^

The initial version of BI-RADS®, dated 1993,^(9)^ was developed by a Committee appointed by Gerald D. Dodd, Jr., M.D., during his tenure as Chairman of the ACR Task Force on Breast Cancer. The ACR Reporting and Data System was divided into 5 sections: I. Breast Imaging Lexicon, II. Reporting System, III. Report Coding System, IV. Pathology Coding System and V. Follow-up and Outcome Monitoring. This initial model contained clinical indication for the exam (screening vs. diagnostic mammography) and persists as one of the most important features in structuring the workflow of U.S. radiologists. The section on Follow-up and Outcome Monitoring highlights the importance of maintaining a database as a quality assurance element of the ACR system. Without systematically monitoring the results of screening, it is impossible to know the success of the program. Each group should maintain the suggested data so that the accuracy of the individual screening programs and their success in diagnosing earlier stage breast cancers can be determined. This will allow each group to adjust its thresholds by comparison with pooled national data.^(9)^

**Figure 2:**
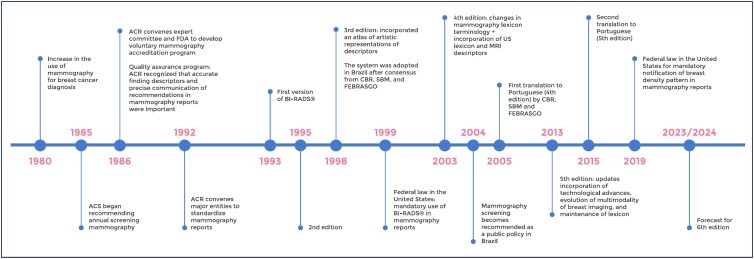
Timeline of events from 1980 to 2024

After its initial creation in 1993, four additional editions were published in 1995, 1998, 2003, and 2013. Each revision of BI-RADS® added important components for clarification, management, and quality assurance.^(9,12–15)^ It was intended to be a "living" document that changes as new data is acquired and new technologies are incorporated.^(2)^ All historical milestones were summarized and highlighted in the timeline, in figure 2.

The second version was published, especially after a systematic review in the results monitoring section of the initial document, to reflect the changes that occurred in mammography in those last few years. A section with all modifications to the original document has been included in detail (Appendix D - release notes with comments: deletion, addition and modification), one of the most important: a letter coding for assessment categories changed to number Categories 0-5.^(12)^

Its third edition (1998)^(13)^ incorporated an Illustrated atlas of artistic representations. The Illustrated BI-RADS® was an extension of the second edition and included illustrations of each feature described, a section on auditing a mammography practice, and sample reports. The third edition of BI-RADS® formally launched data collection for the ACR National Mammography Database (NMD). The ACR elicited participation and encouraged radiology practices to submit data to the NMD, in an attempt to improve interpretive skills of individual radiologists through collection, review, and comparison of their practice data. In addition, the success or failure of the screening program to detect occult cancers at the expected rate could be evaluated and compared with regional and national standards.^(13,16)^

In the 1990s the focus of BI-RADS® was mammography. The fourth edition (2003)^(14)^ revised the terminology of mammographic lexicon (some examples: asymmetry instead of density, "coarse and heterogeneous" and "fine pleomorphic" calcifications), subdivided category 4 findings into a, b, and c, and also incorporated the lexicon of ultrasound and magnetic resonance image MRI) descriptors of the breasts.^(14,16)^

Technological progress, the evolution of multimodality of breast imaging, and the maintenance of lexicon served as the basis for the fifth edition, released in 2013, further facilitating communication between radiologists and requesting physicians.^(16)^ This edition contains over 700 clinical images, updated breast composition descriptors, new descriptors for ultrasound-based elasticity evaluation and magnetic resonance (MR) descriptors for breast implants, follow-up and result monitoring including mammography, ultrasound, and breast MRI, as well as guidance with frequently asked questions for each section (FAQ).^(15,16)^

Other improvements to the BI-RADS® manual include an expanded FAQ section and data tables summarizing published studies that validate descriptors in the lexicon (e.g., the likelihood of malignancy associated with the distribution of calcifications). The BI-RADS® website provides publicly available downloadable reference cards, FAQ documents and selected content from the mammography, ultrasound, and MRI manuals. Open access to website content includes a summary of clinically relevant audits, guidance on data to be collected, and derived data to evaluate.^(17)^ We find numerous relevant information, updates on BI-RADS® and other RADS. Two supplemental publications were incorporated into the 5th edition in the mammography chapter, in 2019 for tomosynthesis^(18)^ and in 2022 for contrast mammography.^(19)^

The sixth edition is expected to be released soon and based on preliminary presentations at international conferences, its content will likely include updates to the three established imaging methods (mammography, ultrasound, and MRI) and implement some novelties, such as detailed indications for exams (screening vs. diagnosis), inclusion of new descriptors, greater emphasis on the lymph node assessment as well as information on tomosynthesis and perhaps contrast mammography in the Atlas.

### BI-RADS® in Brazil

**Figure 3:**
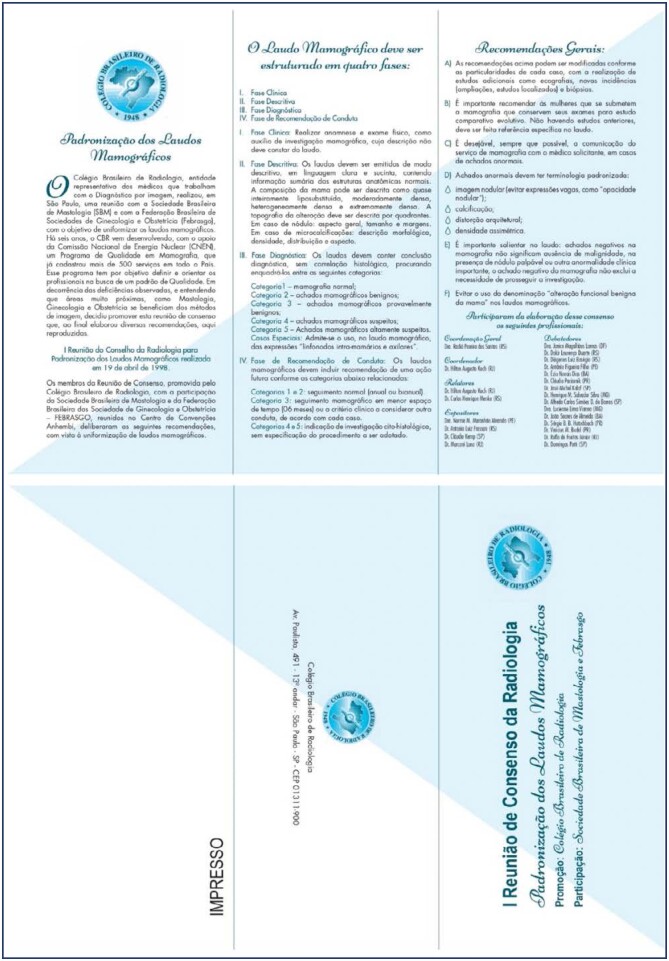
In 1998, the structured report model was adopted in Brazil. Original document

In the early 1990, the Brazilian College of Radiology (CBR), with the support of the National Commission of Nuclear Energy, initiated a Quality Program in Mammography. This movement occurred, just like in the United States, due to deficiencies observed in the quality aspects of mammographic images and medical reports. For this reason, on April 19, 1998, the structured report model was adopted in Brazil after a meeting in São Paulo between the CBR, the Brazilian Society of Mastology (SBM), and the Brazilian Federation of Associations of Gynecology and Obstetrics (FEBRASGO). See attached original document in the figure 3.

In 2004, mammographic screening was recommended as a public policy. Given the relevance of the BI-RADS® system, it has been used by the Ministry of Health and the National Cancer Institute José Alencar Gomes da Silva (INCA) in the development of technical parameters for the programming of breast cancer screening procedures.^(20)^ Finally, in 2012, following the example of the U.S. law of 1992, the National Program for Quality in Mammography (PNQM) was created through a GM/MS Ordinance, updated in 2017. According to the Current Ordinance, PNQM is mandatory for all radiology services that perform mammography in the national territory.^(21)^

In 2005, we had the first translation of BI-RADS® into Portuguese (based on the fourth edition), after a joint effort between CBR, SBM, and FEBRASGO. The work was carried out by a specialized translator, after obtaining the ACR license for the literal translation of the original document. The Portuguese BI-RADS® facilitated access and its dissemination throughout the national territory.^(22)^ The fifth edition (2013) was also translated after its publication, and made available in 2016.

### The importance of BI-RADS® and the evolution of the system to other RADS

Successful breast cancer detection programs in high-income countries can be attributed in part to the widespread use of the American College of Radiology’s Breast Imaging Reporting and Data System (BI-RADS®). In addition to improved communication, BI-RADS® facilitates the monitoring of patient outcomes by providing guidelines concerning how to perform an audit with target performance benchmarks. This allows for a critical "feedback" component necessary to improve the interpretive performance of the radiologist. A high abnormal interpretation rate (e.g., false-positive biopsies) and false-negative results (e.g., missed cancers) require additional radiologist training.^(23)^

**Figure 4:**
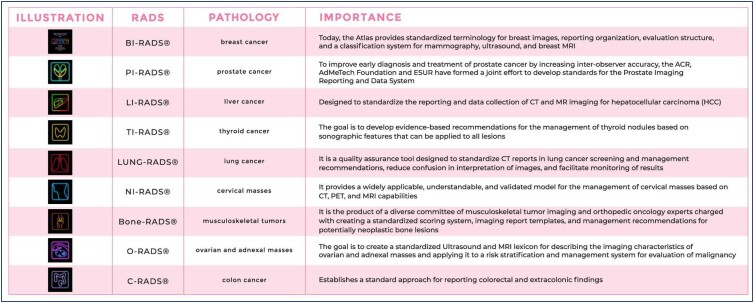
Illustration of the established RADS and their respective importance

With all the success and recognition of the BI-RADS® in standardizing reports on imaging findings, other specialties have adopted the RADS model. Currently, there are 9 systems endorsed by the ACR, and others in development.^(6)^ A brief evaluation of the importance of each system is presented below, summarized in figure 4.

There are 2 collaborative RADS, developed in conjunction with the ACR and other entities: CAD-RADS® and I-TIRADS®. The CAD-RADS® aims to improve communication of results of coronary angiography (CTA) to referring physicians. The societies involved in this consensus were the Society of Cardiovascular Computed Tomography (SCCT), American College of Cardiology (ACC), ACR, and North American Society of Cardiovascular Imaging (NASCI). As for I-TIRADS®, it is a thyroid imaging reporting and data system developed by the International Thyroid Nodule Ultrasound Working Group Steering Committee, in collaboration with the ACR. Other RADS systems in development include PE-RADS® (pulmonary embolism), MSK-RADS® (for soft tissue lesions), Brain Tumor-RADS® (brain tumors), and three more currently being evaluated: KI-RADS® (renal lesions), Stroke-RADS® (for stroke evaluation), and MI-RADS® (molecular imaging).^(6)^

### The use of BI-RADS® in the United States and the particularities of Brazil

#### U.S. Reality

BI-RADS® was developed and is applied within the context of the U.S. healthcare system, marked by strict laws and protocols with standardized procedures. Images and clinical information are electronically recorded and integrated, allowing for correlation between methods and longitudinal analysis, which is essential for the diagnosis of breast pathology. The U.S. technological infrastructure offers much more resources, with state-of-the-art facilities and the latest generation equipment. The population has greater access to information and has the power to demand this access, becoming an active part of the process through civil society organizations, such as the American Cancer Society or Susan G. Komen Breast Cancer Foundation. In addition, there is greater adherence to screening programs. Medical education programs are effective and ensure more assertive conduct, as well as rationing of healthcare resources. In breast radiology, it is the radiologist’s role to interpret the images and issue a report in accordance with the BI-RADS® standardization, which has been mandatory by federal law since April 1999.^(1,24)^ It is often their responsibility to define the next steps for conduct, whether it be guidance on correlation with other imaging exams, follow-up, biopsy or surgery, adjuvant treatment, or returning/proceeding with the screening program. Ultrasound and other imaging exams are complementary or supplemental, and correlation between methods is carried out systematically. Supplemental screening with ultrasound, for example, began to be executed more rigorously after another federal law in 2019,^(25,26)^ which mandates the reporting of breast density patterns in women throughout the country. This systemic organizational dynamic supports the specialist’s work and improves the accuracy of imaging findings expressed in the form of standardized reporting categories, and as a consequence, leads to more assertive conduct.

#### Brazilian Reality

**Figure 5:**
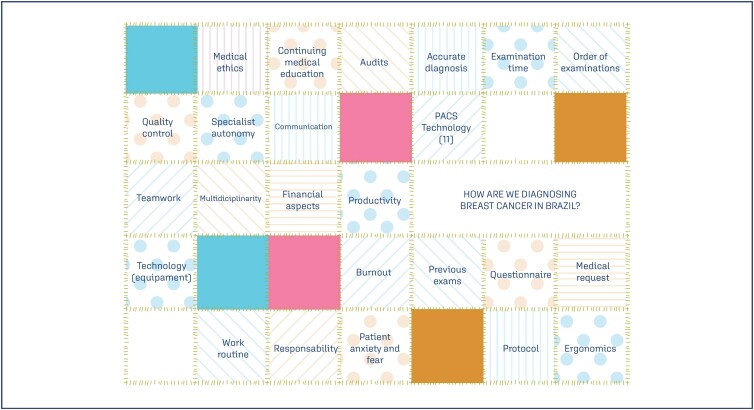
Particularities of Brazil related to the use of the BI-RADS® system

The reality of the Brazilian healthcare system is heterogeneous and presents characteristics that diverge from those where BI-RADS® was developed. We face cultural and regional issues, opportunistic screening, errors in the order of exams, lack of autonomy of specialists in conducting diagnoses, excessive requests for ultrasounds and unnecessary tests. Migowski et al.,^(26)^ cite that between 2014 and 2016, the number of breast ultrasounds performed in women aged between 35 and 69 years in Brazil – data from the Unified Health System (SUS, per Portuguese acronym), was almost three times higher than the number of diagnostic mammograms in the same period and age group. Besides, there are medical requests without the most relevant information and no detailed patient history, patients with little information/orientation, disconnected and unstructured medical file systems, which often result in multiple identification records for the same patient. In addition, images and clinical information are recorded in a decentralized manner, often times not even recorded, and even less electronically. The Picture Archiving and Communication System (PACS) is more present in the major centers of the country. This system is responsible for the evolutionary leap that radiology has witnessed in recent decades, facilitating the correlation between imaging methods, comparison, and evolutionary analysis of cases. The technological park presents variations throughout the national territory, from state-of-the-art equipment, more often available in private clinics in large urban centers, to scrapped devices, notably in the Unified Health System (SUS). From the perspective of professionals, Brazilian medical education and work routine in Brazil differ from the United States and other countries. Breast imaging exams can be interpreted by physicians from different specialties who are often not able to correlate all methods. There is a great shortage of breast radiology specialists in several regions of the country. It is worth noting that deficiencies in training and the lack of mandatory Continuing Medical Education programs can contribute to incorrect conduct and waste of financial resources. For good practice in breast radiology, in most cases, the findings need to be correlated between all imaging methods, comparison with previous exams is fundamental, and the order of exams needs to be preserved. Particularities of the use of BI-RADS® in Brazil are displayed in figure 5.

Regarding BI-RADS®, despite its well-established use and encouragement by the main medical institutions related to breast care in Brazil (CBR, FEBRASGO, and SBM), we face numerous challenges related to our particularities. Concerns about the underutilization of the system throughout the national territory have been highlighted since the beginning of its implementation.^(27)^ We have no official records of audit data, or monitoring of results, which makes it impossible to map the reality of the country.

## Conclusion

The historical analysis of the BI-RADS® from the American College of Radiology (ACR) demonstrates its pioneering role and validates its successful 30-year trajectory worldwide. Its implementation is closely related to the initiative and outcomes of the Mammography Quality Standards Act, a U.S. law dating from 1992. Since its initial creation in 1993, when it was intended only for mammography analysis, the reporting system has evolved to become a comprehensive tool for quality assurance for mammography, ultrasound, magnetic resonance imaging, and more recently, tomosynthesis and contrast-enhanced mammography. The BI-RADS® is a dynamic document evolving with the practice of medicine. Its importance lies in the consistent and appropriate use of lexicon terminology and final assessment categories, with effective communication of imaging findings, malignancy risk estimation, and application of patient and physician recommendations, and providing a system for monitoring results and performing research, data analysis, and auditing. In addition to emphasizing the significance of medical ethics, continuous medical education, and the necessity for comprehensive training in breast imaging, including proficiency in using BI-RADS®, it is imperative to reconsider institutional protocols and the daily work routine of physicians engaged in breast cancer screening and diagnosis. On the eve of the publication of its 6th edition, it is important to recognize how we are using BI-RADS® in Brazil. This topic deserves more attention, detailed and in-depth analysis to identify difficulties, failures, problems, and improvement opportunities.
